# Perceptions of health research participation in rural and urban Pennsylvanians

**DOI:** 10.1017/cts.2026.10718

**Published:** 2026-03-06

**Authors:** Jennifer B. McCormick, Jennifer Mary Poger, Sandesh Bhandari, Courtney Whetzel, Angel Collie, Alyson G. Eggleston, Jennifer L. Kraschnewski, Orfeu M. Buxton

**Affiliations:** 1 Department of Humanities, Penn State College of Medicine, The Pennsylvania State University, Hershey, PA, USA; 2 Penn State Clinical and Translational Science Institute, Penn State College of Medicine, The Pennsylvania State University, Hershey, PA, USA; 3 Department of Medicine, Penn State College of Medicine, The Pennsylvania State University, Hershey, PA, USA; 4 Department of Biobehavioral Health, College of Health and Human Development, The Pennsylvania State University, University Park, PA, USA

**Keywords:** Rural, research participation, motivators, barriers, engagement

## Abstract

**Introduction:**

Emerging evidence describes the experiences of individuals participating in health research, but insights into the barriers and motivations around research participation in rural communities are limited. We developed and administered a human-centered, evidence-informed survey to assess motivators and barriers to research participation among adults in Pennsylvania.

**Methods:**

The online survey captured differences between individuals with and without prior research participation and living in rural and urban settings. We hypothesized that individuals with prior research experience would report different motivators and barriers than those who had never participated in research. We also anticipated that rural and urban respondents would differ in their reported motivators and barriers to participation.

**Results:**

Participants (n = 284, 75% female, 63% urban, 73% with prior research) completed the survey in spring of 2025. Overall top motivators to research participation included a willingness to “contribute to knowledge and medicine,” to “help others,” to “make a difference,” “because the research was personally important,” and “financial compensation.” Top barriers included an “inconvenient research site,” “limited transportation access,” and “time/work constraints.” A variety of motivators and barriers differed by prior research experience. There were no significant differences between the proportion of rural and urban prior research participants who endorsed any of the motivators or barriers. Rural, non-research participants drew greater motivation from “family influence” and “volunteering commitment.”

**Conclusion:**

The results of this work can inform the development of targeted strategies to improve research engagement, particularly among rural populations.

## Introduction

A growing body of research is dedicated to understanding the experiences of individuals participating in health research, including their perspectives regarding the consent process [1], concerns around data sharing and privacy [2], the importance of supportive logistics [3], and the foundational role of trust [4]. Literature consistently demonstrates that research participation is influenced by a complex interplay of personal, social, and structural factors. For example, Kost et al. conducted focus groups with research participants and found that motivations for participation often centered around altruism and the personal relevance of the study, with financial incentives serving as a less important motivator [5]. However, many participants expressed dissatisfaction over not receiving individual or aggregate study results and a growing gap in research translation, a frustration also substantiated in our recent work [6,7]. While altruism and trust enhanced research engagement, barriers such as distrust, logistical challenges, and a lack of clear communication remain significant [5,8,9].

Additionally, rural perspectives on research are likely shaped by health and healthcare experiences differing from their urban counterparts. For example, rural counties in PA have fewer primary care physicians (PCP), medical specialists, and allied health professionals, including five counties without maternal care [10]. Unfortunately, this geographic isolation leads to numerous health disparities, including higher infant mortality rates, as well as higher rates of chronic conditions such as cancer and obesity [11]. Such barriers are further exacerbated in rural communities with limited access to healthcare and research infrastructure due to hospital closures and the absence of other community safety nets [12,13]. National data show disparities in research engagement between rural and urban populations [14], underscoring the importance of understanding the unique motivators for, and barriers to, research participation experienced by rural residents. Existing studies largely rely on qualitative data, focus on specific diseases, or reflect investigator perspectives rather than participant viewpoints [15,16].

We developed and administered a human-centered, evidence-informed survey to assess motivators and barriers to research participation among adults in Pennsylvania. The survey was specifically designed to capture differences between individuals with and without prior research participation and between those living in rural versus urban areas. We hypothesized that individuals with prior research experience would report different motivators and barriers than those who have never participated in research. We also anticipated that rural and urban respondents would differ in their reported motivators and barriers to participation. A key goal of this study is to inform the development of targeted strategies to improve research engagement, particularly among rural populations.

## Methods

### Recruitment and outreach

Participants were recruited by email invitations, website postings, flyers, and word-of-mouth to complete a one-time deidentified survey capturing their perceptions of research and basic demographic characteristics. Names and contact information were collected separately in a post-survey form to facilitate compensation. The most effective method of recruitment for this study was leveraging the Volunteer Repository, a platform to quickly and easily identify studies across Penn State with searchable filters. Built onto the Penn State StudyFinder infrastructure, the Volunteer Repository gives interested volunteers the opportunity to self-select research topics of interest, enhancing their awareness of research opportunities and connecting them with relevant studies through targeted communication. The email invitation described the purpose of the survey in lay terms, provided eligibility criteria (18 years of age+), and estimated participant commitment (approximately 15 minutes). Almost half of the study sample (*N* = 122) was obtained from email invitations to the Volunteer Repository participants. Additionally, a study flyer was created and disseminated via the Penn State University Park and College of Medicine Clinical Research Centers, Penn State Health clinics, and the LION Mobile Clinic, which reaches rural central Pennsylvania communities [17,18].

### Survey methodology

A 33-item questionnaire was developed through previously cited themes around barriers/motivators to research participation [5,19,20]. Five motivation themes emerged and were adapted during survey design: altruism or contribution [5,19,21,22], incentive [21], personal relevance [19,23], relationships, and trust [21,22]. Similarly, six barrier themes were selected or adapted to better understand the needs and perceptions of Pennsylvanians: access [19,24], incentive, ethics [20], health concerns [19,20,24,25], management [19,24], withholding of study outcomes [5,19], and distrust of site or team [20,22].

Questions (Table 1) regarding potential barriers and motivations for participating in research were structured as multiple-choice questions where participants could select all barriers/motivators that applied to them. Each barrier/motivator was then coded as “Yes” or “No” for analysis based on whether the participant selected it or not. The survey was conducted using REDCap, a secure, HIPAA-compliant web application for survey data collection [26,27]. After finishing the survey, participants were asked to complete a separate secure form via REDCap to provide contact information to enter a drawing for one of ten $100 gift cards. All identifying information was collected solely for compensation purposes, and their main survey responses remained deidentified.

**Table 1.** Motivators and barriers to research participation

**Research participants:** “What has motivated you to participate in research (Select all that apply, i.e., yes/no response)? What could be barriers to participating in research for you (Select all that apply, i.e., yes/no responses)?”	
**Non-research participants:** “What could motivate you to participate in research (Select all that apply)? What have you experienced as barriers to participating in research (Select all that apply, i.e., yes/no responses)?”	
**Motivators**	**Barriers**
I want to help others	Challenges with transportation to get to research location
Study topic is relevant to me or my loved ones	Research location is not convenient for me
It’s the only option left to help my condition	Lack of good internet connection
I am committed to volunteering	Missing work or arranging childcare for study visits
Previous positive experiences with the research team	Too much time commitment
I want to contribute to advancing knowledge and medicine	I don’t understand the purpose of the study
Financial compensation	My cultural identity is not reflected in the study team
Free healthcare	Pressure to stay in study
I research this area or a related area	Experiencing pain or extended discomfort
Family Influence	I worry about experiencing side effects
Getting to know the research team better	I have pre-existing conditions that make me ineligible for research studies
Feeling like my participation makes a difference	Payment problems
The research team is trustworthy	Staff is disorganized or unprofessional
The organization providing the research opportunity is trustworthy	Won’t receive clinical test results during the study
Other	Won’t receive study findings
	Have to wait or experience cancellations
	A family member or friend had a bad experience participating in a research study
	Feeling like a human guinea pig
	Surprises or unexpected aspects of the study
	Lack of privacy
	My data may be used against me
	The research team has not demonstrated trustworthiness
	The organization providing the research opportunity has not demonstrated trustworthiness
	I only want to follow to advice of my doctor

### Classification of rurality

Rurality was determined by the state-based definition set forth by the Center for Rural Pennsylvania [28], which is based on population density. Using the Center for Rural Pennsylvania’s definitions, there are 48 rural counties (26% of the state’s population) and 19 urban counties in PA. We intentionally used Pennsylvania’s statutory rurality definition because the study is situated within a state-specific policy and healthcare delivery context. Unlike federal classifications (e.g., USDA), which define rurality as “non-urban,” Pennsylvania’s definition operationalizes rurality directly and is the basis for state health, workforce, and funding decisions. Using this framework therefore aligns the analysis with how rural populations are served in practice. According to the 2020 Census, the population density of Pennsylvania is 291 people per square mile. The Center for Rural Pennsylvania defines a county as rural if the population density of that county is fewer than 291 people per square mile. Participants were asked to provide their zip code in the demographic part of the survey. The zip codes were then mapped onto their corresponding counties. A separate rural vs. urban variable was then created based on county.

### Human participant protections

This survey study received an IRB exempt determination. The authors assert that all procedures contributing to this work comply with the ethical standards of the relevant national and institutional committees on human experimentation and with the Helsinki Declaration of 1975, as revised in 2008. A summary explanation of research was provided to participants at the beginning of the survey. Survey completion was taken as implied consent to participate.

### Statistical analyses

Descriptive statistics were calculated for demographic variables like age, race, and biological sex of the participants. The primary outcome was a binary (Yes/No) variable that indicated a participant’s past participation in research while the primary independent variables were binary (Yes/No) responses related to the different motivators and barriers to research participation. Chi-squared tests of independence were used to explore the relationship between the primary outcome variable and each of the primary independent variables. The results were only considered reliable when the chi-squared test assumptions were fulfilled (i.e., expected cell size > 5). Statistical significance was evaluated at *α* = 0.05.

## Results

### Descriptives of sample

**Figure 1.**
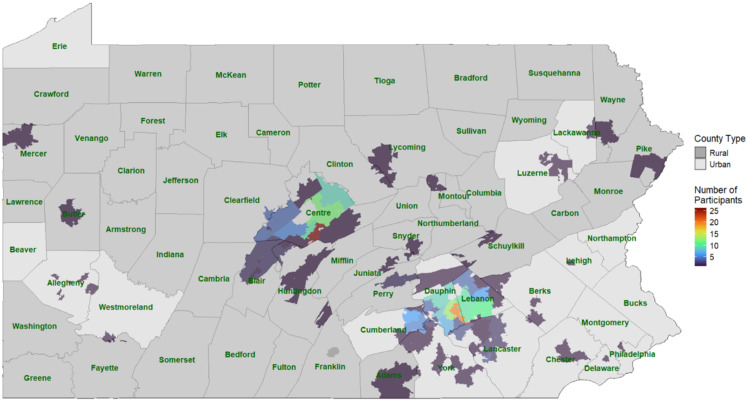
Survey respondent spatial distribution in commonwealth of Pennsylvania. Heat map density by number of participants in zipcode. Rural urban counties are depicted as darker gray, urban counties lighter grey.

Out of 345 completed surveys, 284 reported residing in Pennsylvania and answered the question about prior participation in research. All analyses were performed using data from these 284 participants. Most of the participants were White (81.3%), and 74.6% of participants reported their legal sex at birth to be female (See Table 2). Most (63%) of participants lived in urban areas in Pennsylvania (see Figure 1, Pennsylvania map with locations of study participants).

**Table 2.** Demographics of the survey participants from Pennsylvania

Characteristic	Overall *n* = 284^a^	Previous participation?		County status	
		Participated before *n* = 208^a^	Not participated before *n* = 76^a^	Rural *n* = 105^a^	Urban *n* = 179^a^
**Age group**					
18–24	34 (12.1%)	26 (12.6%)	8 (10.5%)	17 (16.3%)	17 (9.6%)
25–34	45 (16.0%)	28 (13.6%)	17 (22.4%)	15 (14.4%)	30 (16.9%)
35–44	34 (12.1%)	29 (14.1%)	5 (6.6%)	9 (8.7%)	25 (14.0%)
45–54	44 (15.6%)	33 (16.0%)	11 (14.5%)	17 (16.3%)	27 (15.2%)
55–64	55 (19.5%)	36 (17.5%)	19 (25.0%)	15 (14.4%)	40 (22.5%)
65 and over	70 (24.8%)	54 (26.2%)	16 (21.1%)	31 (29.8%)	39 (21.9%)
**Race (multiple choice)**					
Am. Indian	3 (1.1%)	2 (1.0%)	1 (1.3%)	2 (1.9%)	1 (0.6%)
Asian	13 (4.6%)	11 (5.3%)	2 (2.6%)	8 (7.6%)	5 (2.8%)
Black	10 (3.5%)	7 (3.4%)	3 (3.9%)	1 (1.0%)	9 (5.0%)
Hispanic	16 (5.6%)	9 (4.3%)	7 (9.2%)	6 (5.7%)	10 (5.6%)
Middle Eastern	4 (1.4%)	4 (1.9%)	0 (0.0%)	1 (1.0%)	3 (1.7%)
Hawaiian	2 (0.7%)	1 (0.5%)	1 (1.3%)	1 (1.0%)	1 (0.6%)
White	231 (81.3%)	171 (82.2%)	60 (78.9%)	87 (82.9%)	144 (80.4%)
Not listed	1 (0.4%)	1 (0.5%)	0 (0.0%)	1 (1.0%)	0 (0.0%)
Prefer not to answer	11 (3.9%)	8 (3.8%)	3 (3.9%)	3 (2.9%)	8 (4.5%)
**Legal Sex at Birth**					
Female	209 (74.6%)	145 (70.7%)	64 (85.3%)	74 (70.5%)	135 (77.1%)
Male	64 (22.9%)	53 (25.9%)	11 (14.7%)	28 (26.7%)	36 (20.6%)
Prefer not to answer	7 (2.5%)	7 (3.4%)	0 (0.0%)	3 (2.9%)	4 (2.3%)
**Current legal sex**					
Different sexual development or intersex	1 (0.4%)	1 (0.5%)	0 (0.0%)	1 (1.0%)	0 (0.0%)
Female	208 (73.8%)	146 (70.5%)	62 (82.7%)	72 (68.6%)	136 (76.8%)
Male	66 (23.4%)	53 (25.6%)	13 (17.3%)	29 (27.6%)	37 (20.9%)
Prefer not to answer	7 (2.5%)	7 (3.4%)	0 (0.0%)	3 (2.9%)	4 (2.3%)

*Note*: Out of 286 Pennsylvania participants, 2 did not answer the “Participate” question. Therefore, the following tables only includes 284 participants.

a *n* (%).

### Comparisons by prior participation

**Figure 2.**
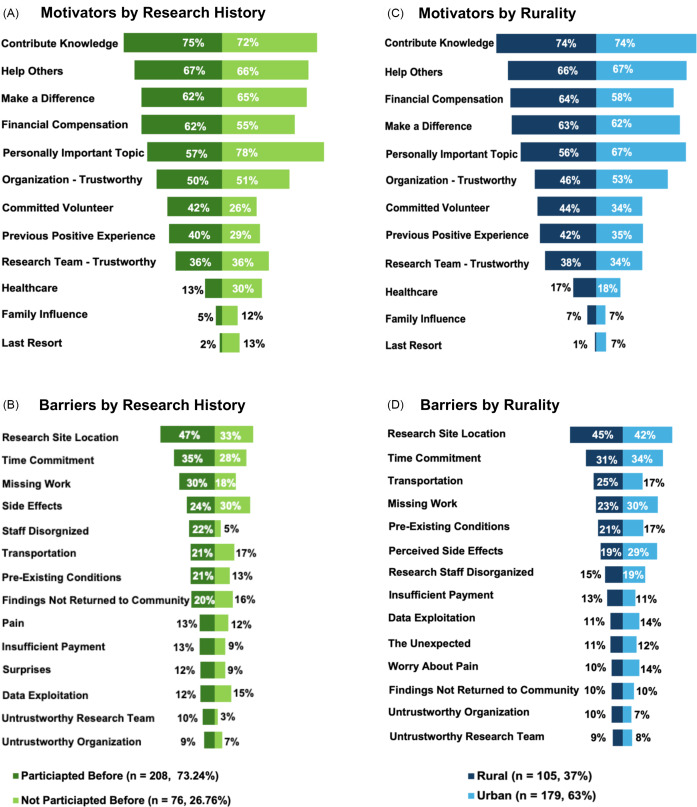
Motivators and Barriers to research participation. (A) Motivators and (B) Barriers to research participation, comparison by research participation history. (C) Motivators and (D) Barriers to research participation, comparison by rurality.

Overall, 208 participants reported prior participation in research while 76 reported having never participated before. Group comparisons of motivators are shown in Table 3. Overall, participants with prior research participation experience were more likely to report a commitment to volunteering as a motivator. Participants without prior participation were more likely to endorse “free healthcare,” “study topic is relevant to me or my loved ones,” and “getting to know the research team better” as motivators. “It’s the only option left to help my condition” was also more likely to be endorsed by participants without any prior research participation, but the data did not meet the requirements for a reliable analysis, so we cannot be confident in this result. The comparisons of reported barriers are shown in Table 4. Overall, participants with prior research participation were more likely to report “staff is disorganized or unprofessional” as a barrier. There were no significant differences between the two groups for any other barrier to research participation. However, there were trends suggesting that participants with prior participation might be more likely to consider “inconvenient research location,” “missing work,” and “research team’s trustworthiness” as barriers. See Figure 2, left panels.

**Table 3.** Motivators for participation in research

Characteristic	Overall *n* = 284^a^	Previous participation?		*p*-Value^b^	County status		*p*-Value^b^
		Participated before *n* = 208^a^	Not participated before *n* = 76^a^		Rural *n* = 105^a^	Urban *n* = 179^a^	
**Motivators (multiple choice)**							
** Contribution**							
I want to help others	189 (66.5%)	139 (66.8%)	50 (65.8%)	>0.9	69 (65.7%)	120 (67.0%)	>0.9
Feeling like my participation makes a difference	177 (62.3%)	128 (61.5%)	49 (64.5%)	0.8	66 (62.9%)	111 (62.0%)	>0.9
I want to contribute to advancing knowledge and medicine	211 (74.3%)	156 (75.0%)	55 (72.4%)	0.8	78 (74.3%)	133 (74.3%)	>0.9
** Incentive**							
Financial compensation	170 (59.9%)	128 (61.5%)	42 (55.3%)	0.4	67 (63.8%)	103 (57.5%)	0.4
Free healthcare	50 (17.6%)	27 (13.0%)	23 (30.3%)	0.001**	18 (17.1%)	32 (17.9%)	>0.9
** Personal relevance**							
Study topic is relevant to me or my loved ones	178 (62.7%)	119 (57.2%)	59 (77.6%)	0.003**	59 (56.2%)	119 (66.5%)	0.11
It’s the only option left to help my condition	14 (4.9%)	4 (1.9%)	10 (13.2%)	<0.001***^c^	1 (1.0%)	13 (7.3%)	0.037*
I research this area or a related area	33 (11.6%)	24 (11.5%)	9 (11.8%)	>0.9	9 (8.6%)	24 (13.4%)	0.3
Family influence	19 (6.7%)	10 (4.8%)	9 (11.8%)	0.067	7 (6.7%)	12 (6.7%)	>0.9
I am committed to volunteering	107 (37.7%)	87 (41.8%)	20 (26.3%)	0.024*	46 (43.8%)	61 (34.1%)	0.13
** Relationship**							
Getting to know the research team better	38 (13.4%)	21 (10.1%)	17 (22.4%)	0.013*	19 (18.1%)	19 (10.6%)	0.11
Previous positive experiences with the research team	106 (37.3%)	84 (40.4%)	22 (28.9%)	0.10	44 (41.9%)	62 (34.6%)	0.3
** Trust**							
The research team is trustworthy	101 (35.6%)	74 (35.6%)	27 (35.5%)	>0.9	40 (38.1%)	61 (34.1%)	0.6
The organization providing the research opportunity is trustworthy	143 (50.4%)	104 (50.0%)	39 (51.3%)	>0.9	48 (45.7%)	95 (53.1%)	0.3
** Other**							
Something else could motivate me to participate in research	19 (6.7%)	17 (8.2%)	2 (2.6%)	0.2	9 (8.6%)	10 (5.6%)	0.5

a *n* (%).

b Pearson’s Chi-squared test.

c Chi-squared approximation may be incorrect because of low expected cell size.

*p* < 0.05; **p < 0.01; ***p < 0.001.

**Table 4.** Barriers to participation in research

Characteristic	Overall *n* = 284^a^	Previous participation?		*p*-Value^b^	County status		*p*-Value^b^
		Participated before *n* = 208^a^	Not participated before *n* = 76^a^		Rural *n* = 105^a^	Urban *n* = 179^a^	
**Barriers (multiple choice)**							
** Access**							
Challenges with transportation to get to research location	56 (19.7%)	43 (20.7%)	13 (17.1%)	0.6	26 (24.8%)	30 (16.8%)	0.14
Research location is not convenient for me	122 (43.0%)	97 (46.6%)	25 (32.9%)	0.053	47 (44.8%)	75 (41.9%)	0.7
Lack of good internet connection	10 (3.5%)	9 (4.3%)	1 (1.3%)	0.4^c^	2 (1.9%)	8 (4.5%)	0.4^c^
Missing work or arranging childcare for study visits	77 (27.1%)	63 (30.3%)	14 (18.4%)	0.066	24 (22.9%)	53 (29.6%)	0.3
Too much time commitment	94 (33.1%)	73 (35.1%)	21 (27.6%)	0.3	33 (31.4%)	61 (34.1%)	0.7
I don’t understand the purpose of the study	22 (7.7%)	15 (7.2%)	7 (9.2%)	0.8	6 (5.7%)	16 (8.9%)	0.5
** Ethics**							
My cultural identity is not reflected in the study team	11 (3.9%)	7 (3.4%)	4 (5.3%)	0.7^c^	4 (3.8%)	7 (3.9%)	>0.9^c^
Pressure to stay in study	24 (8.5%)	16 (7.7%)	8 (10.5%)	0.6	7 (6.7%)	17 (9.5%)	0.5
** Health concerns**							
Experiencing pain or extended discomfort	35 (12.3%)	26 (12.5%)	9 (11.8%)	>0.9	10 (9.5%)	25 (14.0%)	0.4
I worry about experiencing side effects	72 (25.4%)	49 (23.6%)	23 (30.3%)	0.3	20 (19.0%)	52 (29.1%)	0.084
I have pre-existing conditions that make me ineligible for research studies	53 (18.7%)	43 (20.7%)	10 (13.2%)	0.2	22 (21.0%)	31 (17.3%)	0.5
** Incentive**							
Payment problems	33 (11.6%)	26 (12.5%)	7 (9.2%)	0.6	14 (13.3%)	19 (10.6%)	0.6
** Management**							
Staff is disorganized or unprofessional	50 (17.6%)	46 (22.1%)	4 (5.3%)	0.002**	16 (15.2%)	34 (19.0%)	0.5
Won’t receive clinical test results during the study	27 (9.5%)	18 (8.7%)	9 (11.8%)	0.6	10 (9.5%)	17 (9.5%)	>0.9
Won’t receive study findings	53 (18.7%)	41 (19.7%)	12 (15.8%)	0.6	21 (20.0%)	32 (17.9%)	0.8
Have to wait or experience cancellations	22 (7.7%)	17 (8.2%)	5 (6.6%)	0.8	6 (5.7%)	16 (8.9%)	0.5
A family member or friend had a bad experience participating in a research study	12 (4.2%)	10 (4.8%)	2 (2.6%)	0.6^c^	3 (2.9%)	9 (5.0%)	0.6^c^
** Trust**							
Feeling like a human guinea pig	17 (6.0%)	15 (7.2%)	2 (2.6%)	0.2^c^	7 (6.7%)	10 (5.6%)	>0.9^c^
Surprises or unexpected aspects of the study	32 (11.3%)	25 (12.0%)	7 (9.2%)	0.7	11 (10.5%)	21 (11.7%)	0.9
Lack of privacy	28 (9.9%)	22 (10.6%)	6 (7.9%)	0.7	9 (8.6%)	19 (10.6%)	0.7
My data may be used against me	36 (12.7%)	25 (12.0%)	11 (14.5%)	0.7	11 (10.5%)	25 (14.0%)	0.5
The research team has not demonstrated trustworthiness	23 (8.1%)	21 (10.1%)	2 (2.6%)	0.073	9 (8.6%)	14 (7.8%)	>0.9
The organization providing the research opportunity has not demonstrated trustworthiness	23 (8.1%)	18 (8.7%)	5 (6.6%)	0.7	10 (9.5%)	13 (7.3%)	0.7
I only want to follow the advice of my doctor	9 (3.2%)	5 (2.4%)	4 (5.3%)	0.4^c^	2 (1.9%)	7 (3.9%)	0.6^c^
** Other**							
Something else could be a barrier to participating in research for me	32 (11.3%)	23 (11.1%)	9 (11.8%)	>0.9	13 (12.4%)	19 (10.6%)	0.8

a *n* (%).

b Pearson’s Chi-squared test.

c Chi-squared approximation may be incorrect because of low expected cell size.

*p* < 0.05; **p < 0.01; ***p < 0.001.

### Comparisons by rurality

Group comparisons of motivators and barriers by rurality are shown in Tables 3 and 4, respectively. Rural participants were more likely to endorse “It’s the only option left to help my condition” as a motivator. Aside from this, there were no significant differences between the proportion of rural and urban participants who endorsed any of the other motivators or barriers. However, there were trends suggesting that urban participants might be more likely to endorse “worry about experiencing side effects” as a barrier. See Figure 2, right panels.

## Discussion

The unique constellation of challenges, motivators, and barriers faced by rural participants necessitates that their lived realities be made central in any study design involving these communities. We conducted a quantitative survey of motivators and barriers, extracting key findings from prior research participant studies, and adapted to a rural context. Our goal was to surface distinct perspectives from both prior research participants and non-research participants, particularly in rural communities often underrepresented in research engagement. This 33-item REDCap survey included five motivation themes: contribution, incentive, personal relevance, relationships, and trust; and six barrier themes: access, incentive, ethics, health concerns, management, and distrust of site or team. Despite the anticipated skew toward urban participants (63%), the study secured substantial rural representation (37%). Adults who were older than fifty-five (44.0%), white (81.3%), and who identified as female (74.6%) comprised most of the sample. Of interest, women were even more heavily represented in the research participant group (70.7%).

Regardless of prior research participation or rural setting, the top six motivators to research participation included a willingness to “contribute to knowledge and medicine” (74%), to “help others” (67%), “because the research was a personally important topic” (63%), to “make a difference” (62%), “financial compensation” (60%), and “working with a trustworthy organization” (50%). Participants’ motivations support previous work suggesting that altruism[5,19,22,23] and willingness to contribute to meaningful causes[4,19,21] are key facilitators to research participation. Furthermore, personal relevance[19,23] and a trustworthy organization[21,23] have both been shown to increase research participation. This is particularly true when studies align with participants’ own experiences or their communities’ needs[4,19] and are conducted by researchers and organizations perceived to be credible and trustworthy [21,23].

Despite the similarities in the top six motivators to participate in research, some differences based on prior research experience emerged. Participants with prior research experience were more likely to endorse volunteerism as a motivator than their non-research-participating counterparts (42% and 26%, respectively). In contrast, participants without prior research experience were more likely to endorse the following four motivators than those with prior research experience: (1) the study topic is “relevant to me and my loved ones” (78% and 57%, respectively); (2) “free healthcare” (30% and 13%); (3) the opportunity to “get to know the research team better” (22% and 10%); and (4) “it’s the only option left to help my condition” (13% and 2%, a small cell size limiting confidence in the chi-square test). However, for the last motivator, the chi-square assumption of expected cell size greater than 5 was not met when comparing participants with and without prior research experience, limiting confidence in that specific result. This assumption was met when comparing rural and urban participants.

Our findings align with prior research identifying common participant burdens as major barriers to participating in research regardless of prior research experience. An inconvenient research site location emerged as one of the most reported barriers. Several studies have identified research site location as a barrier, often reflecting transportation issues such as limited access to reliable transportation [3,19,29] and inability to afford travel costs [3]. Time constraints and inflexible scheduling also emerged, supporting earlier studies that identified time commitment [3,30,24], work-related conflicts[3,31,24], and other competing commitments such as a lack of child and elderly care[31] as significant barriers to research participation. Further, concern about side effects was an endorsed barrier, which may emphasize participants’ ongoing worries about the perceived risks and safety of participating in research [3,5,3,31]. “Disorganized and unprofessional staff” was one of the most frequently reported barriers by prior research participants, amplifying previous findings that associate this factor with negative research experiences [5]. Overall, our findings emphasize barriers that continue to limit research participation across a range of populations and study contexts.

Differences in overall motivator and barrier patterns across municipality types were insignificant, with rural respondents indicating slightly more trust in research teams and site than urban respondents (38% and 34%, respectively). Rural respondents also were more concerned with the trustworthiness of the research organization than their urban counterparts (53.7% and 45.7%). Respondents without prior participation were more likely to endorse motivators to research participation, including “free healthcare,” “study topic is relevant to me or my loved ones,” and “getting to know the research team better.” Previous work has identified positive relationships with research staff and financial compensation as key factors in continued research participation [5]. Respondents without prior participation may view these identified motivators as anticipated benefits based on their own perceptions of research involvement. Respondents with prior research participation were more likely to report “staff is disorganized or unprofessional” as a barrier; other barriers across cohorts were not significantly different. Urban respondents were more sensitive to convenience, time burden, and financial incentives. These barriers contrast with those from a prior qualitative study of 212 rural and urban participants in a southeastern state [30], which found both rural and urban groups endorsed fear, side effects, lack of understanding of clinical trials, time commitment, and distrust as barriers. These mixed findings highlight the need for future research to better understand how barriers to research participation intersect with the geographic location and the demographic composition of the sample.

While there were no significant differences based on rurality, there were trends suggesting that urban participants might be more likely to endorse “worry about experiencing side effects” as a barrier. Prior work highlights that individuals from rural populations experience significant barriers to research participation[21,32]. Our findings serve as an avenue for future work to inform the development of targeted strategies to improve research engagement, particularly among those who have not participated in research in rural populations.

### Limitations

This study has several limitations. The cross-sectional design and snowball recruitment methods limit the representativeness of the sample and causal inferences that can be drawn. Future studies of rural Pennsylvanians could employ systematic sampling methods that cover more or randomly selected regions/counties to allow for weighted estimations of motivators and barriers to research. It would have been interesting to look at the comparisons between motivators/barriers within subgroups of participants split by rural/urban and previous participation/no prior participation in research. However, the small sample size of rural participants without prior research participation limited our ability to make reliable comparisons within these subgroups. Although informed by qualitative studies, quantitative surveys such as ours are limited in improving understanding on how to address identified research barriers, warranting additional qualitative studies. Future studies could include ratings of the strength of agreement with the motivators/barriers in order to identify the most impactful direction for interventions.

## Conclusion

Enhancing the research experience for rural communities – particularly through strategies that reflect their needs and concerns – is essential to improving participation in research. A positive research experience can also have an outsized impact on participant engagement in future studies.
